# Information Technology and Medical Technology Personnel´s Perception Regarding Segmentation of Medical Devices: A Focus Group Study

**DOI:** 10.3390/healthcare8010023

**Published:** 2020-01-21

**Authors:** David Johansson, Patrik Jönsson, Bodil Ivarsson, Maria Christiansson

**Affiliations:** 1 Department of Information Technology, Region Skåne, 205 25 Malmö, Sweden; patrik.x.jonsson@skane.se; 2 Medical Services, Region Skåne, Malmö 205 25, Sweden; bodil.ivarsson@med.lu.se (B.I.); maria.christiansson@med.lu.se (M.C.); 3 Department of Cardiothoracic Surgery, Lund University, 221 00 Lund, Sweden

**Keywords:** cybersecurity, healthcare technology, patient safety, staff attitudes

## Abstract

*Objective:* Segmentation is one way of improving data protection. The aim of this study was to investigate Information Technology (IT) and Medical Technology (MT) personnel’s perception in relation to ongoing segmentation of medical devices and IT infrastructure in the healthcare sector. *Methods:* Focus group interviews with 9 IT and 9 MT personnel in a county council in southern Sweden were conducted. The interviews focused on two areas: Positive expectations and misgivings. Digital recordings were transcribed verbatim and analyzed using qualitative content analysis. *Results:* Responses related to 2 main areas: Information security and implementation of segmentation. Informants stated that network segmentation would increase the overall level of cybersecurity for medical devices, addressing both insider and outsider threats. However, it would also increase the need for administration and the need for knowledge of the communication patterns of medical devices from the manufacturer’s perspective. *Conclusion:* IT and MT personnel in a county council in southern Sweden believed that segmentation would increase cybersecurity but also increase administration and resource needs, which are important opinions to take into consideration. The present study can be used as a model for others to increase awareness of opinions of healthcare organizations.

## 1. Introduction

Medical Technology (MT) devices as defined in Medical Device Directive (MDD) [1] and Information Technology (IT) serve an increasingly central role in clinical practice, improving patient health, safety, and quality of life. The number of medical devices that are connected to the network are constantly increasing [2,3,4].

Networked medical devices can also cause substantial harm since they have not historically been designed with focus on cybersecurity [5]. Errors that underlie device-related injuries are often categorized into three types: Manufacturer-related errors, use or design errors, and user errors [6]. It has been said that the weakest link in the process is the user, who must understand how to configure and use Medical Technology products correctly to achieve a high level of security in computing infrastructure [7,8].

Cybersecurity is today one of the most important security-related challenges for all countries, but its visibility and awareness are still limited to the public, although almost everyone has heard something about it [9]. There is a substantial security risk posed by outsiders identifying potential interactions between the interconnected elements in hospital systems and computing infrastructures, and taking advantage of poor cybersecurity to steal medical health records, deny access to health services, or cause intentional harm with these [10,11]. In relation to medical devices, it may be possible for hackers to use these in order to gain access to confidential patient data and to reprogram them to send harmful commands [12]. This could mean, for example, altering blood groups or test results, or taking control of pumps that regulate the administration of potent drugs [13]. Likewise, disruption of the parameter values used in the scanning protocols, tampering with the radiation exposure levels, mechanical disruption, and denial-of-service attacks [14].

Other sources have also investigated the subject of cybersecurity in medical devices and have concluded that there is a relationship between the increase in network connectable medical devices and increased cybersecurity risks [15]. Likewise, segmenting the network in multiple layers with security gates such as firewalls in between them could be an effective way to contain network problems and reduce the impact of a breach in network security [16,17], which is comparable to dividing a building into fire zones to delay fire spreading and enabling firefighting in sections rather than in the building as a whole [18].

One way to reduce that risk and prevent intrusion is network segmentation of medical devices. This entails separating elements such as computers, servers, routers, data, and healthcare personnel into groups, thereby restricting access and better protecting vital services [19,20]. Segmentation of medical devices is described as a good method for ensuring data security and is recommended to be used to whatever extent is feasible [21]. In order to increase understanding of innovations in healthcare organizations, such as segmentation of medical devices, Länsisalmi et al. [22] argue that health innovations should be investigated from the perspectives of stakeholders, in this case IT and MT personnel. Therefore, the aim of this study was to investigate IT and MT personnel’s perception in relation to segmentation of medical devices and IT infrastructure in the healthcare sector.

Highlights

- Medical devices can cause substantial harm.
- The user of medical devices must understand how to achieve a high level of security.
- Hackers might be able to reprogram medical devices to send harmful commands.
- One way to reduce the risk of hackers and prevent intrusion is network segmentation.
- MT and IT personnel must be positive to the increase in cybersecurity.

## 2. Material and Methods

### 2.1. Design and Setting

A qualitative methodology design was employed with focus group sessions in order to gain a deeper insight into the views and experiences of IT segmentation. This descriptive study was conducted at Region Skåne, one of the 21 county councils/regions in Sweden. Region Skåne employs around 34,000 people, mostly in healthcare, and has the main responsibility for public healthcare and medical services in the region [23]. Much of the healthcare is conducted in a high-tech environment, which includes devices such as patient monitoring equipment, medical imaging systems, and laboratory instrumentation. A number of cybersecurity-related incidents prompted a decision to implement network segmentation for all devices in Region Skåne. The study was performed during the ongoing implementation of the network segmentation of medical devices.

### 2.2. Informants

Informants were strategically recruited from IT and MT personnel based on their experience working with medical devices, such as patient monitoring equipment, medical imaging systems, and laboratory instrumentation in Region Skåne, to ensure maximal variation in socio-demographic data and service positions [24]. The following variables were taken into account to ensure a broad selection: Age, time in the profession, and competence in (1) segmentation, (2) MT device systems, (3) firewall-based traffic filtering, and (4) IT security. 

### 2.3. Ethics

The study conformed to the principles outlined in the Declaration of Helsinki and according to Swedish legislation (SFS 2003:460) no ethical approval was required as the study did not involve patients. The project was approved by the directors of the participating departments. All informants were informed of the aims and procedures of the study through a short written presentation included in an email invitation. Informed consent was considered implied when informants signed up voluntarily for the focus group interviews. The data processing was carried out in accordance to the General Data Protection Regulation (GDPR)

### 2.4. Data Collection

Three mixed MT and IT focus group interviews [25] were held with 5 to 8 informants in each group and included a total of 18 informants (9 MT personnel and 9 IT personnel, see Table 1 for demographic and other characteristics). Interviews were conducted in a conference room and took about 90 min each. The discussions were digitally recorded (with the informants’ consent) and independently transcribed by a trusted agency.

**Figure 1:**
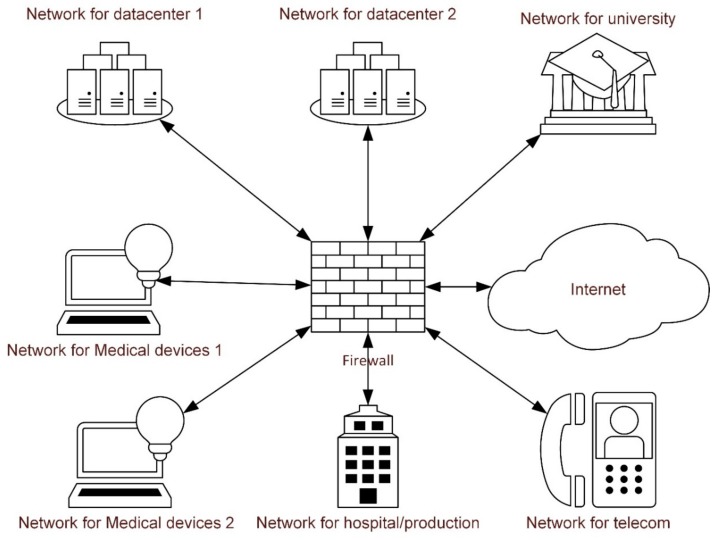
Example of rough outline of network segmentation.

The interviews were conducted with one researcher who had extensive experience in focus group interviews acting as moderator and a second acting as assistant. A short presentation of network segmentation was held prior to discussions as an introduction to the topic. A rough outline of network segmentation and specifically their relationship to the segment of medical devices was also presented (see Figure 1). The interviews focused on two different areas, i.e., positive expectations and misgivings, and informants were asked two open-ended questions: “What are the advantages of segmentation of medical devices?” and “What misgivings do you have regarding segmentation of medical devices?” Probing questions were also used. 

### 2.5. Data Analysis

The text from the interviews was analyzed using qualitative content analysis [26,27]. Transcribed interviews were first read as a whole by all authors to gain an overall understanding. The text was then divided into meaningful units, which were condensed, coded, and gathered into groups with similar content. These steps were carried out by the authors individually and then all authors gathered and discussed the findings. These were then abstracted this into subcategories that met the study aim. Finally, the subcategories were abstracted into two categories. Example of the analysis process can be found in Table 2. The statements were thoroughly assessed by the authors to ensure that no statements relating to positive expectations or misgivings regarding segmentation were left out due to the condensation of meaning units into codes.

## 3. Results

Two categories emerged during the analysis of the transcribed interviews: “Information security” and “Implementation of segmentation in healthcare”. These categories contained six subcategories (Table 3).

### 3.1. Information Security

#### 3.1.1. Outsider Threats

Across the three focus groups, attacks originating from outside of the healthcare organization were well known and the informants had personal experience of such attacks or had heard of them from others. Virus attacks had been experienced that led to numerous problems and increased work. Informants were on the whole positive to the possibilities of limiting data virus infections that are offered by segmentation technology and thus increase patient safety. They were also positive to the increased protection that segmentation offered to older applications unable to withstand outsider threats due to patching policies or lack of security design. 

It was stated that segmentation reduces the risk that core systems or patients will be affected during an infection of malicious software or hacker attacks/intrusions, by restricting access between technical devices.

“*The thing is, that you can divide up and protect…When you look at virus attacks and other things, that it doesn’t affect different segments but stays within the same segment…That’s the advantage as I see it*” (Informant 3)

#### 3.1.2. Insider Threats

In relation to insider threats, informants had experience of personnel accidentally forwarding infected emails that caused great harm. They perceived that segmentation would reduce the risk of users getting hold of restricted information via administrative interfaces that they should not have access to, thereby reducing the probability of them making administrative mistakes. It also would reduce the probability that users, whether by mistake or intention, introduced malicious code into medical devices and/or core infrastructure. The informants opined that with segmentation devices would be less likely to be incorrectly configured when connected to the network, thereby reducing the likelihood of failures or connection issues and, in turn, improving patient security. *“…It doesn’t have to be malicious, of course, you might make a mistake just because you have access to things that you shouldn’t have.”* (Informant 2). 

However, the informants expressed there was a risk that so many openings would be needed between segments due to the large numbers of older devices that the benefits of segmentation would be cancelled out.

### 3.2. Implementation of Segmentation in Healthcare

#### 3.2.1. Predictions of an Increase in Costs and Administration

The informants, in general, predicted an increase in administration and costs for the organization. Because of the higher level of complexity introduced by segmentation, the informants predicted that working with a segmented network would require better planning and documentation. A positive side effect of this would be a better understanding of future costs for the healthcare organization, which would be especially useful for detecting increased costs due to deviating or nonstandard network communication solutions for Medical Technology. It was also expected that segmentation would lead to better planned maintenance work for Medical Technology solutions since network communication would be more controlled and structured.

The informants assumed that implementing segmentation would increase administrative tasks and the need for resources (time consuming) due to the increased numbers of firewalls between segments, and openings needed in these. Also, new processes, routines, and administrative tools would be needed, such as automated software. There were, therefore, misgivings that segmentation would lead to longer execution times and that increased planning would be required for network changes.

“*I see it [implementation] as very resource-intensive to implement…longer lead times and complicated administration.*” (Informant 17)

#### 3.2.2. Predictions of Required Knowledge Improvement for the Healthcare Organization

The informants perceived that their own knowledge and competence regarding network communication would need to improve, due to a higher level of complexity. Informants had positive expectations of receiving the necessary education and assumed that management felt the same way.

However, the informants also stated the importance of informing all relevant parties about the strategy and goal of the network segmentation to, e.g., personnel affiliated with system management and Medical Technology devices within the healthcare organization.

“*I see an advantage and a disadvantage in the fact that more knowledge is required…not only from us, but also from our suppliers…With segmentation, we will need a better understanding of all of our systems.*” (Informant 1)

#### 3.2.3. Possibilities and Pitfalls Concerning Segmentation

The informants perceived that the segmentation solution provided an opportunity to use the infrastructure to provide restricted communication rights based on actual needs of users and systems, which would provide a high degree of flexibility and thereby better preparedness for new communication requirements. It also presented an easier way to reuse the infrastructure. They also expressed that the design gave an advantage by isolating attacks through the network in an easier way, and that it gave the basis for a more controlled and secure network operations environment by reducing the impact of changes and faults between network segments. Furthermore, they said that dedicated network segments for remote administration would give fewer attack vectors between systems.

The informants highlighted the need to communicate overall policies and designs, as well as guidelines for implementation of segmentation, to avoid counterproductive effects. One such effect could be creating too many openings between the segments so that the key benefits of segmentation would be lost.

“*Segmentation is really about trying to minimize consequences…If we discover that one segment has a virus…then we shut down the framework, which means that it’s completely isolated.*” (Informant 18)

#### 3.2.4. Increased Demands on Medical Device Manufacturers

The informants had experience of working with medical device manufacturers who connect to medical devices remotely. It was foreseen that requirements for knowledge, planning ability, and documentation from manufacturers of how their devices work and how they integrate with other devices would increase once the segmentation is completed. This would give increased insight into devices, something that informants perceived as currently lacking, both in customers and manufacturers. However, this could also lead to increased cost.

“*…And then I think another problem could be that our suppliers, they don’t always know exactly how their applications talk to each other, which ports are used. So that could also be challenging to get right.*” (Informant 15)

Segmentation was also expected to give an opportunity for manufacturers to connect to devices for support in more diverse and secure ways. For instance, it would be possible to allow forms of communication other than virtual private network (VPN) without affecting the overall security level. It would also be possible to reduce manufacturer’s access rights to other systems, thus reducing the risk of manufacturer-induced faults. (Informant 5)

## 4. Discussion

This qualitative study provides insights into the perceptions among IT and MT personnel after a decision to implement network segmentation of medical devices and IT infrastructure in the healthcare sector. Despite that this is a small study, the results corroborated that the healthcare systems are complex structures, and medical devices that are integrated into a hospital’s IT systems are just a minor part of the system as a whole. However, these devices play a critical role in clinical function. If a medical device fails, there are many potential hazards to patient safety. There are many techniques for increasing cybersecurity, including not only network segmentation but also security policies, antivirus software, lifecycle management of operating systems, security patches, drivers, and so on [15]. Hopefully, the present study may partly provide support and inspire healthcare organizations to develop and communicate a strategy that encompasses all these important parts. Generally, security can be further enhanced if the segmentation is implemented with rigorous and strictly enforced security controls regarding communication between segments. As such, the communication boundaries between segments will not stop cyberattacks entirely unless the network also complies with rules, standards, and requirements that emphasizes access control policies using so called “next generation firewall” (NGFW) implementation which includes additional network device-filtering functionalities such as intrusion prevention system (IPS), intrusion detection protocol (IDP), activity logging, and web and mail security features. A simple application layer firewall cannot stop camouflaged malware from causing security risks to healthcare facilities.

In this study, which focused specifically on expectations and misgivings regarding segmentation, there were two themes that emerged across the categories identified in the analysis, namely information security and implementation of segmentation in healthcare. The informants in the study had experience of insider and outsider threats. In May 2017, a major ransomware attack called WannaCry occurred, which affected 200,000 systems in around 150 countries worldwide [13]. In the UK, 50 hospitals were affected, patient safety was threatened, and there were calls to highlight cybersecurity in healthcare [13]. In the present study, informants assumed that insider and outsider threats and threats from malicious software, such as ransomware, would decrease with the use of segmentation. Besides the advantages pointed out by the informants, segmentation adds additional security measures to hinder attack vectors. An example is the possibility to deny certain types of network traffic on layer 7 of the Open Systems Interconnection (OSI)-stack (i.e., message handling in the application layer), which allows detection of communication patterns. This will decrease the spread of malicious code and denial-of-services threats. Medical devices are regarded to have a low capability of defending themselves against these sorts of threats and will benefit greatly from the added security layers. It could be speculated that if all England's hospitals had implemented segmentation of medical equipment ahead of the of WannaCry attack [13], the impact would probably have been lessened as the dispersion could have been limited. If this were the case, hospitals could have continued to produce care and thus maintained a higher level of patient safety, especially if there had been support for technologies, such as intrusion prevention system (IPS). However, healthcare organizations need to be aware that segmentation, although important, is just one measure to increase cybersecurity; relying on it solely could give a false sense of security.

Informants in the study opined that with segmentation, the demand for documentation would increase regarding communication between infrastructure components within and between systems. It was also opined that the number of firewall changes (i.e., allowed network traffic patterns between segments) and the overall workload of the network and system administrators would increase, which, in turn, would lead to technical skills and increased costs. These opinions are important to take into account from a change management perspective as the informants represent a critical success factor in achieving a high cybersecurity level. Underinvesting in cybersecurity is not unique for Sweden. It has previously been pointed out that compared to other sectors that spend 4–10%, of its budget, the healthcare sector spends a relatively small amount (1–2%) of its budget on IT infrastructure. This must be reconsidered so that fundamental patient safety, healthcare technology, and functionality are not compromised [13,28]. It can be stated that, as healthcare organizations are relying more and more on digitalization, both regarding medical and other systems, the need for cybersecurity will also become more aligned with other sectors, such as industries and banks.

On the basis of the present study, the suggestion of a more structured way of working can be perceived as less flexible with regard to changes, from an administrative point of view, and require new processes and new supporting software. On the positive side, segmentation will give better insight into the communication landscape, and a better overview. Historically, cyberattacks have had a significant impact on production, generating high costs for the targeted organization, and the healthcare sector must find cost-effective ways to prevent such potentially devastating costs [13]. It must also be assumed that the confidence of stakeholders (patients, manufacturers, and institutions) in the healthcare organization will be reduced after a successful cyberattack.

Our results showed a need for improvement in the level of competence as the informants assumed that a greater knowledge base would be required due to the higher level of complexity associated with segmentation. This is in line with a Swedish study showing that a majority of IT and MT personnel considered recurrent training on IT and MT safety, rules, and regulations, as well as a risk analysis with a focus on patient safety, to be of great importance [7]. It is also important that the goals and importance of segmentation are communicated throughout the organization, and the processes and guidelines established regarding system placement within the different segments. The healthcare organization must commit to and supply the means for education and training.

The informants in this study assumed that segmentation would provide more efficient and flexible ways of working for healthcare personnel since changing network sockets will not result in additional administrative tasks. Also, dedicated segments for remote administration add better control and logging capabilities in relation to external users, such as medical device suppliers providing support. The informants also identified a great need for thorough planning before the implementation of segmentation, to make the administration, configuration, and traffic analysis manageable for the organization. The number of segments increases the amount of hardware required, and it is also important to plan where medical devices are placed to minimize administration between segments.

In order to meet the needs of cybersecurity of the future, the present study stated that the knowledge level of medical device manufacturers, in general, needed to be increased in association with segmentation. It has earlier been highlighted that increased collaboration between medical device manufacturers and personnel in the healthcare sector is fundamental to ensure effective protection [29]. One effect of segmentation is that suppliers will gain new possibilities to connect to their medical devices within the healthcare organization, for example, during support cases. Segmentation will not reduce the regulation and security requirements that suppliers need to commit to. This is in agreement with [30] that stated the manufactures had challenges to meet regulation and safety standards including lifecycle management to improve medical device cybersecurity. It is, therefore, important that requirements regarding connection for remote maintenance and support are clearly agreed to during the acquisition process for new medical devices.

We believe mixed IT/MT focus groups increased coverage of the subject area and provided openness in discussions regarding to being better able to extract novel insights and various expert knowledge. However, because of the study’s qualitative nature, the aim was not to generalize the results to the healthcare sector as a whole. However, it is reasonable to assume that the results show a part of the reality of healthcare in one county council as well as give a better understanding of cybersecurity issues. A review on cybersecurity stated that there are very few studies in the area which include human, organizational aspects, strategy, and management. This may justify the present study [31]. A question we have asked ourselves is whether the outcome would have been different if the interviews had been held in the near future after an attack like the WannaCry ransomware attack [13]. That attack is one more indication that cybersecurity policies must be in place for proactive use in the healthcare section and widely communicated, even if the cost seems high. Further, a large (country-scale) study of the implementation of network segmentation would be of interest for further research, and also a study of the health economic impact of a cyberattack.

## 5. Conclusions

Network segmentation decreases the probability of spreading malicious software and intrusions through a network by introducing barriers between network areas, much in the same way fire zones decreases the probability for fire spreading in a building. Medical devices have historically not had a focus on security features. In this study, it was apparent that MT and IT personnel were positive to the increase in cybersecurity provided by network segmentation but concerned about the increase in the administration that it will entail for medical devices. These opinions are important to take into account to be able to reach the desired increase in cyber security. Cyber security risks are multifaceted. The present study can be used as a model for other clinical healthcare manufacturers to increase awareness of concerns and opinions in personnel and healthcare organizations in general. 

## Figures and Tables

**Table 1:** Demographic data and other characteristics.

Gender	
Women	3
Men	15
**Age**	
Mean Standard deviation	52 ± 10
Median (range)	51 (33–67)
**Time in professions (years)**	
Mean SD	18 ± 12
Median (range)	17 (2–40)
**Highest level of education**	
High school (*n*)	3
University (*n*)	15

**Table 2:** Example of the analysis process, from verbatim meaning unit to category.

Meaning Unit	Condensed	Code	Subcategory	Category
…So theoretically, if somebody would have a bad intent, it’s very easy today. Considering that today the risk is greater internally than externally. And it doesn’t have to be malicious, it may be that you make a mistake because you have access to things that you should not have access to. And then that’s really a good thing then, to segment, we clearly get what’s stealing from and we can follow it up in a new way… (Participant 2)	“…it doesn’t have to be malicious, of course, you might make a mistake just because you have access to things that you shouldn’t have” (Participant 2).	Improving patient security	Insider threats	Information Security
“I think it will require a great deal of resources to get it implemented. I see it as very resource-intensive to implement… longer lead times and complicated administration. There is, as we stated, not enough resources to handle requests in firewall changes…” (Participant 17)	“I see it [implementation] as very resource-intensive to implement … longer lead times and complicated administration” (Participant 17)	Resource-intensive project	Predictions of an increase in costs and administration	Implementation of segmentation in healthcare

**Table 3:** Distribution of categories and subcategories concerning Information Technology (IT) and Medical Technology (MT) personnel views in relation to segmentation of medical devices and IT infrastructure in the healthcare sector (*N* = 18).

Categories	Sub-Categories
Information Security	Outsider threats
Implementation of segmentation in healthcare	Insider threats
	Predictions of an increase in costs and administration
	Predictions of required knowledge improvement for the healthcare organization
	Possibilities and pitfalls concerning segmentation
